# Optimal ratio of laurate and butyrate from glyceride forms improves nutrient digestibility and health in weaned piglets

**DOI:** 10.5713/ab.250645

**Published:** 2025-11-14

**Authors:** Shuang Dong, Yi Chen, Nan Zhang, Jihua Wang, Yu Cao, Yongxi Ma

**Affiliations:** 1 State Key Laboratory of Animal Nutrition, College of Animal Science and Technology, China Agricultural University, Beijing, China; 2 Galido Biotechnology Co., Ltd., Wuhan, China

**Keywords:** Antioxidant Capacity, Growth Performance, Immunological Capacity, Medium and Short-chain Fatty Acids, Nutrient Digestibility, Weaned Piglets

## Abstract

**Objective:**

The study aimed to evaluate the effects of varying blend ratios of dietary α-glycerol monolaurate (GML) and glyceryl tributyrate (TB) on growth performance, antioxidant capacity, and immune function in weaned piglets.

**Methods:**

A total of 120 weaned piglets (Duroc×[Landrace×Yorkshire], initial body weight 6.87±0.15 kg, 28 days old) were assigned randomly to three treatments with five replicate pens per treatment for the 28-day experiment. The treatments consisted of a basal diet supplemented with 0.1% GML/TB blend at the following ratios: 1) higher GML (HM; GML/TB = 7:3); 2) balanced ratio (BR; GML/TB = 1:1); 3) lower GML (LM; GML/TB = 3:7).

**Results:**

Dietary BR supplementation increased apparent total tract digestibility (ATTD) of crude protein and gross energy (GE, p<0.05) on day 14 and ATTD of GE on day 28 compared with other groups. Compared with the LM group, piglets fed the BR diet had higher (p<0.05) concentration of alanine aminotransferase (ALT) and interleukin (IL)-1β and lower (p<0.05) levels of diamine oxidase on day 14 had higher (p<0.05) concentration of IL-1β and lower (p<0.05) concentration of IL-6 on day 28 in serum. Dietary BR supplementation increased (p<0.05) the ALT content, decreased (p<0.05) the IL-6 content on day 14 and aspartate aminotransferase and IL-1β contents on day 28, decreased (p<0.05) the IL-10 contents on day 28 in serum compared with the HM group. Furthermore, dietary BR supplementation increased (p<0.05) the activities of glutathione peroxidase in the duodenum, total antioxidant capacity in the jejunum, and catalase in the ileum. Compared with the LM group, piglets fed another two diets had lower (p<0.05) level of malondialdehyde in the duodenum, jejunum, and ileum.

**Conclusion:**

In conclusion, dietary supplementation with a 0.1% GML/TB blend (1:1) improves nutrient digestibility, enhances intestinal antioxidant capacity, modulates inflammatory responses, and supports overall health in weaned piglets.

## INTRODUCTION

Following the prohibition of antibiotic growth promoters, researchers have extensively investigated novel bioactive feed additives. Medium and short-chain fatty acids (MSCFAs), as one of the more effective ones, have garnered significant interest. The biological properties and mechanisms of action of fatty acids are influenced by their carbon chain length [1]. Short-chain fatty acids (SCFAs), which are naturally produced in the intestinal tract of animals, are believed to regulate microbial community and mediate host-microbe interactions [2,3]. Dietary supplementation with SCFAs can be directly absorbed by the intestines and serve as an efficient energy source for animals [4,5]. Medium-chain fatty acids (MCFAs) have also demonstrated efficacy as dietary supplements for piglets, exhibiting antibacterial and growth-promoting properties [6,7]. Studies have shown that individual supplementation of either MCFAs or SCFAs in diets can improve nutrient absorption [8,9]; modulate intestinal barrier function [5,10]; enhance antibacterial and anti-inflammatory abilities [11] in piglets. Our previous study demonstrated that 0.1% MSCFA (α-glycerol monolaurate [GML] and glyceryl tributyrate [TB]) supplementation enhanced nutrient digestibility, immune and antioxidant capacity, and intestinal health in weaned piglets [12]. Similarly, Dahmer et al [13] reported that dietary formulations combining formic acid (SCFA) and GML can significantly improve weaned piglet growth, attributable to immune modulation, and gut microbiota alterations. Furthermore, blends of MCFAs have been observed to have a synergistic effect in promoting growth performance, reducing diarrhea, and inhibiting the growth of harmful bacteria [14,15]. Although these findings are promising, the optimal fatty acid ratio requires further investigation. Based on previous research in our laboratory [12], we established three experimental groups (higher GML [HM], balanced ratio [BR], and lower GML [LM]) with progressively decreasing GML concentrations to identify the optimal ratio. This experimental design enables systematic comparison of different fatty acid ratios on growth performance, nutrient digestibility, antioxidant capacity, and immune function in weaned piglets.

## MATERIALS AND METHODS

### Animals and experimental designs

A total of 120 healthy weaned piglets (Duroc×[ Landrace× Yorkshire], initial BW 6.87±0.15 kg on 28 days old) were assigned randomly to three treatments with five replicate pens (eight piglets per pen, four female and four male) per treatment based on body weight for the 28-day experiment. The treatments consisted of a basal diet supplemented with 0.1% GML/TB blend at the following ratios:1) HM (GML/TB = 7:3); 2) BR (GML/TB = 1:1); 3) LM (GML/TB = 3:7). The additives, comprising GML (900 g/kg) and tributyrin (TB, 600 g/kg), were obtained from by Galido Biotechnology.

The corn-soybean meal basal diets were formulated according to National Research Council [16] nutrient requirements, with detailed composition presented in Table 1. The study was conducted in an environmentally controlled nursery facility, where temperature was maintained at 26°C–28°C and relative humidity at 55%–70%. All piglets had free access to both feed and water throughout the experimental period. Feeding management included twice-daily trough inspections (08:30 and 15:30) to ensure continuous feed availability. Daily monitoring protocols encompassed recording of feed consumption, fecal characteristics, and general health status of all animals.

### Sample collections

Fresh fecal matter from each replicate pen was gathered on days 12–14 and 26–28 of the trial and promptly stored at −20°C. Utilizing a spot sampling method, roughly 400 g of excreta per collection period was obtained, then subjected to oven drying (65°C for 72 h). To facilitate precise subsequent moisture analysis, the desiccated samples underwent a 24-hour equilibration period at ambient conditions. Finally, all fecal specimens were pulverized to achieve particle size passage through a 1mm screen sieve for further analysis.

On the morning of days 15 and 28, five fasted piglets (12 h fasting period) were randomly selected (one per pen) for blood collection [5]. Jugular venipuncture was performed to draw 10 mL of blood into heparin-treated vacuum tubes. Samples were centrifuged (3,000×g, 10 min, 4°C) to harvest serum, which was subsequently stored at −20°C.

On day 28, five piglets approximating the median body weight were selected per treatment group for euthanasia. Segments (2 cm length) from duodenal, jejunal, and ileal mid-regions were excised following luminal content removal and saline rinsing. These intestinal tissues underwent 24-hour fixation in 4% paraformaldehyde for morphological assessment. Mucosal layers from duodenum, jejunum, and ileum were gently harvested using sterile glass slides (Taizhou Huien Medical Equipment) into 1.5 mL centrifuge tubes. Mucosal specimens were preserved at −80°C pending digestive enzyme analysis.

### Growth performance

Individual body weights of piglets were recorded on days 0, 14, and 28 to determine average daily gain (ADG). Daily feed consumption was monitored to compute average daily feed intake (ADFI) and feed conversion ratio (FCR, calculated as ADFI/ADG).

### Chemical analysis for diet and feces

Diet and fecal samples were subjected to analysis for dry matter (DM), ether extract (EE), ash, and crude protein (CP), adhering to the standard methodologies of the AOAC International [17], gross energy (GE) was ascertained utilizing an automatic isoperibol oxygen bomb calorimeter (Parr 1281, Automatic Energy Analyzer), organic matter (OM) was calculated as OM = 100–ash. The acid insoluble ash (AIA) was performed according to the procedure established by Dong et al [12]. Apparent total tract digestibility (ATTD) of dietary nutrients was determined using the following equation:


(1) $$\begin{array}{l} \text{ATTD of nutrients }(\%)=\\ 100-({\text{AIA}}_{\text{diet}}\times {\text{Nutrient}}_{\text{feces}})/({\text{AIA}}_{\text{feces}}\times {\text{Nutrient}}_{\text{diet}})\times 100 \end{array}$$

### Serum physiological and biochemical properties

Serum concentrations of total antioxidant capacity (T-AOC; Catalog No. A015-2-1), malonaldehyde (MDA; Catalog No. A003-1-2), superoxide dismutase (SOD; Catalog No. A001-3), glutathione peroxidase (GSH-Px; Catalog No. A005-1), aminotransferase (ALT; Catalog No. C009-2-1), catalase (CAT; Catalog No. A007-1-1), alanine aspartate aminotransferase (AST; Catalog No. C010-2-1), albumin (ALB; Catalog No. A028-2-1), alkaline phosphatase (ALP; Catalog No. A059-3-1), immunoglobulin A (IgA; Catalog No. H108-1-1), immunoglobulin G (IgG; Catalog No. H106-1-1), and immunoglobulin M (IgM; Catalog No. H109-1-1) were quantified using enzyme-linked immunosorbent assay (ELISA) kits (Nanjing Jiancheng Bioengineering Institute), following the manufacturer’s protocols. Additionally, interleukin-10 (IL-10; Catalog No. H009-1-2), tumor necrosis factor-α (TNF-α; Catalog No. H052-1-2), and interleukin-6 (IL-6; Catalog No. H007-1-1) levels in serum were determined with commercial ELISA kits from the same supplier. Analytical procedures for all assays were performed in accordance with the methodology detailed by Dong et al [12].

### Analysis of intestinal index

Frozen intestinal tissue samples (approximately 400 mg) were accurately weighed and homogenized in 1 mL of ice-cold physiological saline. The homogenates were heated in a boiling water bath (100°C) for 10 min with subsequent homogenization. The samples were then centrifuged at 3,000×g for 15 min at 4°C. The clear supernatants were collected for analysis. The activities/levels of T-AOC, SOD, MDA, GSH-Px, and CAT were assayed using specific commercial ELISA kits (Nanjing Jiancheng Bioengineering Institute) in strict accordance with the manufacturer’s instructions, which were identical to the protocols used for serum samples. The biochemical data were normalized to the protein concentration of the supernatant.

### Intestinal mucosal digestive enzyme activity

The enzymatic activities of amylase (AMS; Catalog No. C016-1-1; Starch-Iodine Colorimetric Method), chymotrypsin (Catalog No. A080-3-1; Colorimetric Method), lipase (LPS; Catalog No. A054 - 1; Colorimetric Method), and trypsin (Catalog No. A080-2; Ultraviolet Colorimetric Method) were determined using assay kits (Nanjing Jiancheng Institute of Bioengineering). All experimental procedures were rigorously carried out in accordance with the manufacturer’s protocols. Sample preparations included homogenization, centrifugation, and appropriate dilution to ensure that absorbance readings fell within the linear range of the respective assays. Enzyme activity was normalized by protein concentration (U/mg).

### Statistical analysis

Data were analyzed using SAS 9.4 (SAS Institute). Normality of residuals and homogeneity of variances were verified by the UNIVARIATE procedure. Growth performance data (ADG, ADFI, FCR) were analyzed with pen as the experimental unit (n = 5 replicates per treatment), while serum parameters, intestinal morphology, and digestive enzyme activities used individual pig as the experimental unit (n = 5 piglets per treatment). A one-way ANOVA model was applied with treatment (HM, BR, LM) as the fixed effect:


(2) $${\text{Y}}_{\text{ij}}=\mu +{\text{T}}_{\text{i}}+{ɛ}_{\text{ij}}$$

where Y_ij_ = observed variable, μ = overall mean, T_i_ = treatment effect, and ɛ_ij_ = residual error.

Multiple comparisons were performed using Tukey’s test, and the results were presented as mean values±standard error of the mean. Significant differences were considered at p<0.05, whereas 0.05≤p<0.10 was considered as a tendency.

## RESULTS

### Growth performance

The growth performance of weaned piglets is presented in Table 2. No statistically significant variations were observed in body weight, ADG, ADFI, or FCR across the dietary treatments (p>0.05).

### Apparent total tract digestibility of nutrients

As presented in Table 3, the ATTD of CP and GE was significantly improved (p<0.05) on day 14 in piglets fed the BR diet compared to other dietary groups. A higher ATTD of GE was also observed in this group on day 28 (p<0.05). Compared with the HM and BR groups, supplementation of the diet with LM decreased (p<0.05) the ATTD of DM and CP on day 28.

### Serum biochemistry indexes

Supplementation of the diet with BR increased the contents of ALT on d 14 in the serum relative to the other two treatments (p<0.05; Table 4) and decreased the level of DAO compared with the LM group. Additionally, the activity of AST in the BR and LM groups was higher (p<0.05; Table 4) compared to the HM group on day 28.

### Serum immune function and inflammatory factors

On day 14, piglets fed BR diet exhibited significantly higher (p<0.05; Table 5) IL-1β concentrations relative to the LM group. Meanwhile, both the BR and LM groups showed lower IL-6 (p<0.05; Table 5) and higher IgM (p<0.05; Table 5) concentrations compared to the HM group. On day 28, dietary HM supplementation resulted in increased IgM and IL-10 levels (p<0.05; Table 5), along with decreased TNF-α and IL-6 concentrations (p<0.05; Table 5), when compared to the LM group. Furthermore, supplementation of the diet with BR in piglets significantly reduced IL-6 while increasing IL-1β concentrations compared with the LM group (p<0.05; Table 5).

### Intestinal status antioxidant

In the duodenum, piglets fed BR diet exhibited significantly higher GSH-Px activity and lower MDA levels compared with the LM group (p<0.05; Table 6). In the jejunum, compared with the LM group, dietary BR supplementation decreased the contents of MDA, while increasing the contents of T-AOC and dietary HM supplementation decreased the contents of SOD and T-AOC (p<0.05; Table 6). In the Ileum, dietary BR supplementation increased the contents of CAT compared with other two groups. Supplementation of the diet with HM and BR decreased the MDA and T-AOC concentration while increasing SOD concentration compared with the LM group (p<0.05; Table 6).

### Digestive enzymes

Compared with the LM group, dietary BR supplementation decreased the activity of chymotrypsin in the duodenum (p<0.05; Table 7) while increasing the activity of trypsin in jejunum. And compared with HM group, piglets fed BR diet had higher activity of trypsin in ileum (p<0.05; Table 7).

## DISCUSSION

MSCFAs not only serve as an energy source [5] but also enhance body health through their unique bioactivity, such as antimicrobial, antiviral, and anti-inflammatory activities [18]. However, most existing studies have focused on individual fatty acids or mixtures of specific fatty acids with other compounds, with limited investigation into the effects of their mixing ratios on piglet growth performance and health. Therefore, based on preliminary data in our laboratory, this study was designed to systematically evaluate the effects of varying GML:TB ratios on weaned piglet performance.

Fan et al [19] found that dietary supplementation with a high content of MCFAs (caprylic acid, capric acid, and lauric acids) improved ADG compared with high content SCFA. Consistent with this, our results showed that dietary supplementation with a HM content (HM and BR group) improved the growth performance of piglets compared with the LM group, especially BR group. This may be attributed to the regulatory effect of these fatty acid blends on the intestinal barrier function, thereby enhancing the absorption efficiency of nutrients [19,20]. Kwon et al [21] showed that a high level of MCFA (caproic and caprylic acid) can increase the FCR without affecting the feed intake. GML supplementation upregulates lipid metabolism related gene expression in piglets, thereby enhancing both lipid metabolism and nutrient absorption efficiency [9]. In present study, supplementing HM (HM and BR group) significantly increased the ATTD of DM, GE, and CP on day 28. This trend was consistent with the growth performance. Therefore, the present study suggests that an appropriate blend ratio (GML:TB = 1:1) likely exert positive effects on growth performance by improving nutrient digestibility.

Serum biochemical indices provide crucial insights into the overall metabolic status and health of animals. ALT and AST serve as key enzymatic markers in amino acid metabolism and both are primarily located within hepatocytes [22]. To some extent, elevated enzymatic activity in the liver may reflect enhanced metabolic efficiency of proteins and lipids, which is often associated with improved growth performance [23]. In the present study, however, the increased serum ALT levels observed in piglets fed the BR diet generally indicate heightened hepatic metabolic activity or potential hepatocellular stress. This elevation in serum ALT activity coincided with improved CP digestibility. It is possible that the specific fatty acid ratio in BR diet enhanced protein metabolism and anabolic processes, thereby increasing the metabolic load on the liver. Therefore, while the rise in ALT may reflect improved protein utilization contributing to growth promotion, it could also signal potential stress on hepatic function. Further investigation is warranted to determine whether this dietary regimen imposes a metabolic burden on the liver. Intestinal barrier not only selectively absorbs nutrients, but also acts as an important defense mechanism, effectively preventing harmful bacteria and toxins from transferring to other tissues [24]. Increased intestinal permeability permits DAO, normally localized in intestinal mucosal cells, to enter systemic circulation. Consequently, plasma DAO concentration serves as a clinical biomarker for intestinal permeability assessment [25]. The results showed that the content of DAO in the serum of piglets fed with the BR diet was significantly reduced on day 14. This indicates that an appropriate ratio of the blend of GML and TB may improve nutrient absorption and reduce pathogenic translocation through intestinal barrier stabilization [24].

Immunoglobulins, including IgG, IgM, and IgA constitute the principal components of humoral immunity, which can provide defense against bacterial and viral pathogens [19]. Dietary supplementation with a functional fatty acid blend increased the concentration of IgM in serum [26]. Butyric acid can increase the number of IgA^+^ plasma cells in the jejunum of piglets, thereby raising the concentration of serum IgG [27]. In the current study, dietary BR supplementation promoted the levels of IgG and IgM in serum on day 28. These results are consistent with the findings of Ma et al [28], who reported that that organic acid blends enhance late-stage (day 15 to 28) immunoglobulin production (IgG, IgM, IgA). Ren et al [29] indicated that supplementing with a 1% mixture of formic acid and propionic acid could reduce the inflammatory response in weaned piglets while improving their immune function. Wang et al [26] have demonstrated that the addition of functional fatty acids can effectively reduce the levels of TNF-α, IL-1β, and IL-8 [26]. In our study, the BR diet reduced the concentrations of IL-10 and IL-6, but paradoxically increased IL-1β levels. This result is partially consistent with previous studies [26,29]. This discrepancy may reflect variations in experimental fatty acid composition ratios. Mechanistically, the entry of butyrate into the cells can inhibit the activation of NF-κB, thereby downregulating the expression of inflammatory factors [30]. MCFA can effectively regulate the expression of cytokines (IL-6, IL-1) in the colon, alleviating local inflammation [31]. In summary, the BR diet modulates immune responses through pleiotropic mechanisms, maintaining systemic low-grade inflammation in piglets.

Post-weaning diarrhea (PWD) is a multifactorial disorder, and while oxidative stress is not considered its primary cause, it frequently emerges as a secondary response to other key contributors (e.g., intestinal barrier disruption, pathogenic colonization, or inflammatory reactions) involved in PWD pathogenesis [32]. This secondary redox imbalance can further impair intestinal barrier integrity, increase intestinal permeability, and thereby facilitate the translocation of pathogenic microorganisms, exacerbating PWD severity [19,32]. Antioxidant enzymes such as SOD, GSH-Px, and T-AOC are the main components of antioxidant stress, while MDA is a marker for measuring oxidative stress. In the current study, the MDA levels of piglets in the HM and BR groups were reduced, while the GSH-Px activity in the BR group was increased. These results demonstrate that optimal fatty acid blends can effectively eliminate excessive free radicals and strengthen antioxidant defense. Studies have shown that sodium butyrate can effectively reduce the MDA level in chicken serum [33], MCFAs, such as lauric acid, exhibit significant antioxidant properties in weaned piglets [34]. Moreover, the combined supplementation of MCFA and butyrate can synergistically enhance the antioxidant capacity of weaned piglets [35]. Overall, current evidence establishes that fatty acid formulations significantly enhance animal antioxidant defenses. Short-chain organic acids (e.g., format, acetate) maintain GSH homeostasis by preventing precursor deficiency induced synthesis impairment, while upregulating GSH-Px activity through enhanced feedback mechanisms, ultimately accelerating radical scavenging [28]. It is plausible that mixed fatty acids regulate the expression profile of antioxidant enzymes through similar molecular mechanisms, thereby enhancing the overall antioxidant capacity of the organism. Therefore, a suitable ratio of mixed fatty acids (GML: TB = 1:1) is helpful in improving the antioxidant capacity of piglets.

Growth performance enhancement shows a significant positive correlation with digestive enzyme activity. Early-weaned piglets exhibit inadequate gastric acid secretion, resulting in suboptimal digestive enzyme activity along the gastrointestinal tract due to inappropriate pH conditions. Supplementation of an acidified fatty acid mixture in the diet can improve the gastrointestinal environment, activate pepsin, promote the secretion of trypsin, thereby increasing feed digestibility and accelerating protein breakdown [36]. In our study, a low proportion of TB (LM group) can significantly increase the trypsin activity in the duodenum, while an equal proportion of GML and TB (BR group) can enhance the trypsin activity in the jejunum and ileum. Dietary GML and TB are metabolized by gut microbiota into laurate and butyrate, respectively, modulating intestinal pH and consequently enhancing digestive enzyme activity [9,37]. Furthermore, MSCFAs can be directly absorbed by intestinal epithelial cells due to their easy absorption properties. These fatty acids stimulate enzymatic secretion through villus height augmentation and potentiate membrane-bound enzyme activity [38]. In conclusion, supplementation of GML and TB enhances the activity of digestive enzymes, and its mechanism of action may be related to the optimization of the intestinal pH environment.

## CONCLUSION

The present study has confirmed that dietary supplementation with mixed fatty acids enhances nutrient digestion and absorption in piglets through improved intestinal barrier function and elevated digestive enzyme activity, leading to enhanced production performance. Additionally, mixed fatty acids improve antioxidant capacity and mitigate inflammatory responses, thereby supporting overall health. Overall, when the ratio of GML to TB is 1:1, piglets can achieve the best growth performance and maintain the optimal health status.

## Figures and Tables

**Table 1:** Detailed composition basal diets (as-fed basis, %)

Items	Content (%)
Ingredients	
Corn	63.48
Soybean meal (43%)	17.00
Extruded soybean	5.00
Soybean oil	1.50
Fish meal	5.00
Whey powder	4.00
CaHPO_4_	1.00
Limestone	0.70
L-lysine hydrochloride (78%)	0.50
L-threonine (98%)	0.20
DL-methionine (98%)	0.07
L-tryptophan (98%)	0.05
Choline chloride (98%)	0.30
Diatomite	0.20
NaCl	0.50
Premix^1)^	0.50
Total	100.00
Nutrient levels^2)^	
Metabolizable energy (MJ/kg)	14.20
Crude protein (%)	18.51
Ether extract (%)	3.49
Dry matter (%)	89.83
Organic matter (%)	84.21
SID lysine (%)	1.28
SID threonine (%)	0.78
SID methionine (%)	0.37
SID tryptophan (%)	0.21

1) The premix provided the following per kilogram of diet: vitamin A, 12,000 IU; vitamin D3, 2,000 IU; vitamin E, 30 IU; vitamin K3, 3 mg; vitamin B1, 3 mg; vitamin B2, 10 mg; vitamin B6, 6 mg; vitamin B12, 24 μg; nicotinic acid, 30 mg; D-pantothenic acid, 30 mg; folic acid, 2 mg; biotin, 0.3 mg; choline chloride, 600 mg; Fe, 120 mg; Cu, 10 mg; Mn, 35 mg; Zn, 120 mg; I, 0.3 mg; Se, 0.3 mg.

2) Metabolizable energy was calculated according to NRC [16]. All others are measured values.

SID, standardized ileal digestible.

**Table 2:** Effects of different ratios of MSCFA on growth performance in weaned piglets

Items	Treatments			SEM^1)^	p-value

	HM	BR	LM		
Body wt 0 d (kg)	6.87	6.87	6.87	1.12	1.00
Body wt 14 d (kg)	11.07	11.26	11.13	1.62	0.97
Body wt 28 d (kg)	17.06	17.41	16.76	2.73	0.91
Day 1 to 14					
ADG (g/d)	300.00	313.57	304.28	40.50	0.88
ADFI (g/d)	480.48	470.51	474.24	68.11	0.80
FCR	1.60	1.52	1.56	0.07	0.31
Day 14 to 28					
ADG (g/d)	427.85	439.28	402.14	92.55	0.84
ADFI (g/d)	693.15	684.88	683.28	154.50	0.99
FCR	1.62	1.56	1.70	0.13	0.24
Day 0 to 28					
ADG (g/d)	363.92	377.21	353.48	62.80	0.86
ADFI (g/d)	586.82	577.70	578.76	121.95	0.99
FCR	1.61	1.53	1.63	0.14	0.47

HM = 0.1% MSCFA (GML/TB = 7:3); BR = 0.1% MSCFA (GML/TB = 1:^1)^; LM = 0.1% MSCFA (GML/TB = 3:7).

1) n = 5.

MSCFA, medium- and short-chain fatty acid; SEM, standard error of the mean; ADG, average daily gain; ADFI, average daily feed intake; FCR, feed conversion ratio; GML, α-glycerol monolaurate; TB, tributyrate.

**Table 3:** Effects of different ratios of MSCFA on nutrient apparent digestibility in weaned piglets

Items	Treatments			SEM^1)^	p-value

	HM	BR	LM		
Day 14					
DM (%)	78.00	81.96	78.79	1.88	0.09
OM (%)	82.14	85.06	82.86	1.76	0.05
CP (%)	67.35^b^	75.36^a^	68.49^b^	3.45	0.02
EE (%)	40.02	41.53	43.51	5.65	0.06
GE (%)	77.21^b^	82.88^a^	78.24^b^	2.04	0.04
Day 28					
DM (%)	79.32^a^	81.69^a^	75.71^b^	2.39	<0.01
OM (%)	83.51	84.05	83.31	1.92	0.21
CP (%)	70.36^a^	71.87^a^	64.58^b^	3.57	<0.01
EE (%)	49.03	48.32	49.14	1.41	0.15
GE (%)	75.06^b^	78.64^a^	74.37^b^	2.56	<0.01

HM = 0.1% MSCFA (GML/TB = 7:3); BR = 0.1% MSCFA (GML/TB = 1:1); LM = 0.1% MSCFA (GML/TB = 3:7).

1) n = 5.

a,b Mean values within a row with different letters differ at p<0.05.

MSCFA, medium- and short-chain fatty acid; SEM, standard error of the mean; DM, dry matter; OM, organic matter; CP, crude protein; EE, ether extract; GE, gross energy; GML, α-glycerol monolaurate; TB, tributyrate.

**Table 4:** Effects of different ratios of MSCFA on serum biochemical indicators in weaned piglets

Items	Treatments			SEM^1)^	p-value

	HM	BR	LM		
Day 14					
AST (U/L)	33.39	47.89	37.61	23.84	0.56
ALT (U/L)	36.75^b^	46.66^a^	38.54^b^	6.48	<0.01
BUN (mg/dL)	5.58	5.85	5.81	1.74	0.86
ALB (g/L)	8.92	8.44	7.54	2.13	0.82
TC (mmol/L)	0.66	0.99	0.91	0.32	0.12
GLU (mmol/L)	3.97	4.44	4.02	0.74	0.70
DAO (U/mL)	1.99^ab^	1.32^b^	2.44^a^	0.48	0.03
Day 28					
AST (U/L)	25.67^b^	41.24^a^	45.45^a^	10.87	<0.01
ALT (U/L)	32.41	41.85	51.29	13.04	0.07
BUN (mg/dL)	6.66	5.56	7.04	2.10	0.20
ALB (g/L)	10.22	11.61	8.77	2.45	0.54
TC (mmol/L)	1.20	1.47	1.46	0.39	0.35
GLU (mmol/L)	4.04	4.36	3.64	1.12	0.22
DAO (U/mL)	2.17	2.37	2.83	1.26	0.36

HM = 0.1% MSCFA (GML/TB = 7:3); BR = 0.1% MSCFA (GML/TB = 1:1); LM = 0.1% MSCFA (GML/TB = 3:7).

1) n = 5.

a,b Mean values within a row with different letters differ at p<0.05.

MSCFA, medium- and short-chain fatty acid; SEM, standard error of the mean; AST, aspartate aminotransferase; ALT, alanine aminotransferase; BUN, blood urea nitrogen; ALB, albumin; TC, total cholesterol; GLU, glucose; DAO, diamine oxidase; GML, α-glycerol monolaurate; TB, tributyrate.

**Table 5:** Effects of different ratios of MSCFA on serum immune function and inflammatory factors in weaned piglets

Items	Treatments			SEM^1)^	p-value

	HM	BR	LM		
Day 14					
Immune indexes					
IgG (g/L)	16.52	18.63	17.35	2.59	0.21
IgM (g/L)	2.06	2.47	1.92	0.30	0.64
IgA (g/L)	0.92	0.81	1.04	0.17	0.38
Inflammatory indexes					
TNF-α (pg/mL)	36.73	38.56	38.01	3.74	0.63
IL-1β (pg/mL)	32.10^ab^	36.91^a^	26.20^b^	5.46	<0.01
IL-6 (pg/mL)	112.91^a^	101.32^b^	102.66^b^	5.92	<0.01
IL-10 (pg/mL)	16.21	14.31	15.36	2.94	0.14
Day 28					
Immune indexes					
IgG (g/L)	19.27	24.03	19.01	4.00	0.09
IgM (g/L)	3.83^a^	2.52^ab^	1.96^b^	1.09	0.01
IgA (g/L)	1.34	1.05	1.38	0.43	0.24
Inflammatory indexes					
TNF-α (pg/mL)	43.29^b^	46.38^ab^	54.06^a^	6.99	0.04
IL-1β (pg/mL)	26.00^b^	33.25^a^	22.81^b^	4.83	<0.01
IL-6 (pg/mL)	102.34^b^	118.06^b^	130.31^a^	14.21	<0.01
IL-10 (pg/mL)	22.63^a^	19.54^b^	18.49^b^	3.23	0.04

HM = 0.1% MSCFA (GML/TB = 7:3); BR = 0.1% MSCFA (GML/TB = 1:1); LM = 0.1% MSCFA (GML/TB = 3:7).

1) n = 5.

a,b Mean values within a row with different letters differ at p<0.05.

MSCFA, medium- and short-chain fatty acid; SEM, standard error of the mean; IgG, immunoglobulin G; IgM, immunoglobulin M; IgA, immunoglobulin A; TNF-α, tumor necrosis factor-α; IL-1β, interleukin-1beta; IL-6, interleukin-6; IL-10, interleukin-10; GML, α-glycerol monolaurate; TB, tributyrate.

**Table 6:** Effects of different ratios of MSCFA on intestinal antioxidant capacity in weaned piglets

Items	Treatments			SEM^1)^	p-value

	HM	BR	LM		
Duodenum					
CAT (U/mL)	18.25	19.03	18.37	3.32	0.97
GSH-Px (U/mL)	314.68^b^	380.46^a^	308.83^b^	50.08	0.03
MDA (nmol/mL)	3.21^b^	3.47^b^	4.98^a^	0.99	<0.01
SOD (U/mL)	47.37	48.30	48.21	4.61	0.94
T-AOC (U/mL)	7.75	8.32	5.86	1.53	0.07
Jejunum					
CAT (U/mL)	33.64	27.03	34.96	7.61	0.07
GSH-Px (U/mL)	353.18	364.89	359.79	40.02	0.61
MDA (nmol/mL)	2.01^b^	1.89^b^	3.32^a^	0.78	<0.01
SOD (U/mL)	43.59^b^	66.95^a^	54.71^a^	8.95	<0.01
T-AOC (U/mL)	8.50^b^	10.33^a^	8.34^b^	1.64	<0.01
Ileum					
CAT (U/mL)	17.03^b^	22.63^a^	16.78^b^	3.83	<0.01
GSH-Px (U/mL)	420.27	364.19	383.37	50.07	0.21
MDA (nmol/mL)	2.77^b^	2.73^b^	4.23^a^	0.85	<0.01
SOD (U/mL)	55.35^a^	53.61^a^	45.08^b^	4.81	<0.01
T-AOC (U/mL)	5.19^c^	7.24^b^	8.08^a^	1.35	<0.01

HM = 0.1% MSCFA (GML/TB = 7:3); BR = 0.1% MSCFA (GML/TB = 1:1); LM = 0.1% MSCFA (GML/TB = 3:7).

1) n = 5.

a,b Mean values within a row with different letters differ at p<0.05.

MSCFA, medium- and short-chain fatty acid; SEM, standard error of the mean; CAT, catalase; GSH-Px, glutathione peroxidase; MDA, malonaldehyde; SOD, superoxide dismutase; T-AOC, total anti-oxidant capacity; GML, α-glycerol monolaurate; TB, tributyrate.

**Table 7:** Effects of different ratios of MSCFA on intestinal mucosal digestive enzymes in weaned piglets

Items	Treatments			SEM^1)^	p-value

	HM	BR	LM		
Duodenum					
AMS (U/mg)	2.83	2.06	2.78	0.98	0.13
Trypsin (U/mg)	1,964.95	1,931.52	2,055.83	513.57	0.85
Lipase (U/L)	66.76	86.31	73.51	15.41	0.09
Chymotrypsin (U/mg)	6.74^ab^	4.97^b^	8.47^a^	2.84	0.04
Jejunum					
AMS (U/mg)	1.47	1.45	2.35	0.82	0.16
Trypsin (U/mg)	2,306.44^ab^	3,024.81^a^	1,925.50^b^	626.31	0.01
Lipase (U/L)	62.06	73.46	61.20	20.51	0.64
Chymotrypsin (U/mg)	2.72	3.78	4.51	1.40	0.16
Ileum					
AMS (U/mg)	1.78	1.65	1.94	0.43	0.25
Trypsin (U/mg)	2,907.29^b^	3,433.65^a^	3,209.37^ab^	307.15	0.04
Lipase (U/L)	83.31	74.28	61.17	18.16	0.19
Chymotrypsin (U/mg)	4.50	4.63	4.72	0.82	0.81

HM = 0.1% MSCFA (GML/TB = 7:3); BR = 0.1% MSCFA (GML/TB = 1:1); LM = 0.1% MSCFA (GML/TB = 3:7).

1) n = 5.

a,b Mean values within a row with different letters differ at p<0.05.

MSCFA, medium- and short-chain fatty acid; SEM, standard error of the mean; AMS, amylase; GML, α-glycerol monolaurate; TB, tributyrate.

## Data Availability

Upon reasonable request, the datasets of this study can be available from the corresponding author.
